# Performance Evaluation of a Microhybrid Dental Restorative Composite Reinforced with Organoclay Nanoparticles

**DOI:** 10.3390/polym18091059

**Published:** 2026-04-27

**Authors:** Alexandros K. Nikolaidis, Konstantinos Ioannidis, Dimitris S. Achilias, Elisabeth A. Koulaouzidou

**Affiliations:** 1 Division of Dental Tissues’ Pathology and Therapeutics (Basic Dental Sciences, Endodontology and Operative Dentistry), School of Dentistry, Aristotle University Thessaloniki, 541 24 Thessaloniki, Greece; 2 Laboratory of Polymer and Color Chemistry and Technology, Department of Chemistry, Aristotle University Thessaloniki, 541 24 Thessaloniki, Greece

**Keywords:** dental restorative materials, microhybrid restorative composites, clay nanoparticles, nanodentistry, montmorillonite

## Abstract

Dental restorative resins available today still have limitations that may affect their durability. This study explores reinforcing a universal microhybrid dental composite resin with organomodified nanoclay at low filler loadings (0, 0.5, 1, 3, and 5 wt%). The morphology, structural features, and light transmittance of the composites were analyzed using scanning electron microscopy (SEM), X-ray diffraction (XRD), attenuated total reflection–Fourier transform infrared (ATR–FTIR), and UV–Vis spectroscopy. The degree of conversion and polymerization shrinkage were measured with ATR–FTIR and a linear variable displacement transducer (LVDT). Water sorption and solubility parameters and flexural properties were assessed gravimetrically and with a dynamometer, respectively. The composites mainly showed exfoliated structures and an improved degree of conversion. Polymerization shrinkage and solubility were lower than those of unmodified dental resin. The highest degree of conversion was observed in composites with 0.5–1 wt% nanoclay. The incorporation of 1 wt% nanoclay resulted in the lowest shrinkage and sorption, along with the highest flexural modulus and strength. Overall, the results suggest that low nanoclay concentrations can improve the physicochemical and mechanical properties of dental composites, highlighting their potential to develop advanced restorative materials that can address current clinical challenges.

## 1. Introduction

The term nanodentistry refers to the application of nanotechnology principles across various dental fields, including prevention, diagnosis, restoration, endodontics, periodontics, prosthodontics, orthodontics, dental tissue bio-mimetics, osseointegration, oral medicine, and maxillofacial surgery [1,2]. By taking advantage of the large surface-to-volume ratio, the use of nanoparticles can produce dental materials with superior mechanical, physicochemical, and biological properties compared to conventional ones. Examples of nanotechnology in modern dental research include remineralizing dental composites containing hydroxyapatite nanoparticles [3], antibacterial endodontic bioceramic sealers reinforced with silver nanoparticles [4], 3D-printed denture-based resins with improved mechanical properties modified with nanoglass particles and multiwalled carbon nanotubes [5], and nanoporous titanium surfaces that enhance osseointegration [6].

Clay nanoparticles have been extensively studied in polymer nanocomposites because of their outstanding mechanical [7], antimicrobial [8], thermal [9], and moisture barrier properties [10]. They are known to be biocompatible at higher concentrations than other nanomaterials, with their degradation products being non-toxic and osseo-inductive [11]. Clay minerals, also called phyllosilicates, consist of tetrahedral (SiO_2_) and octahedral (AlO_6_) sheets arranged in 1:1 (SiO_2_-AlO_6_) and 2:1 (SiO_2_-AlO_6_-SiO_2_) structures. In smectic clays (2:1 types), negative charges on the surface, caused by isomorphous cation substitutions in their sheets, are balanced by exchangeable cations (Na^+^ or Ca^2+^) in the interlayer gallery [11], which can further be replaced by other inorganic or organic intercalant cations to serve specific purposes. Over time, nanoclay particles have garnered interest in various fields of dental research, as the incorporation of calcium, fluoride, silver, zinc, and quaternary ammonium ions (QAIs) into the clay’s interlayer space can enhance the remineralization ability and the antibacterial properties of clay-based dental materials [12]. For example, montmorillonite (MMT) modified with cetylpyridinium chloride (CPC) can enhance the efficacy of mouthwash formulations against oral bacteria [13]. Additionally, the mechanical strength of nanoclay-reinforced glass ionomer cements (GICs) has been improved without impairing the acid–base neutralization reaction [14]. Poly(methyl methacrylate) (PMMA)–organoclay nanocomposites used as denture base materials exhibit increased flexural strength and fracture toughness [15]. Dual-cure composite cements modified with CPC–MMT particles exhibit antibiofilm activity without compromising mechanical strength and bonding performance [16]. Pit and fissure sealants containing fluoride–MMT filler, which are non-cytotoxic, exhibit good fluoride ion release and recharge capability, helping to prevent dental caries [17].

Dental composite resins are bisphenol A-glycidyl methacrylate (Bis-GMA)-based matrix restorative materials reinforced with inorganic fillers, which are favored for their aesthetics and clinical performance. However, the commercially available dental restorative resins still exhibit specific limitations associated with mechanical stability, polymerization shrinkage, water sorption and solubility, biocompatibility, caries inhibitory activity, and marginal leakage, which may compromise their long-term clinical performance [18,19]. To address these drawbacks, dental composites have occasionally been reinforced with Se–ZnO nanoparticles [20], quaternary ammonium polyethyleneimine nanoparticles (QPEIs) [21], TiO_2_ nanofillers [22], and hydroxyapatite nanorods [23], or have been modified with arylophosphazene molecules [24], showing remarkable antibacterial and mechanical properties. Undoubtedly, most of the above reinforcing agents are produced via time-consuming chemical processes demanding organic solvents and high amounts of energy. On the contrary, clay nanoparticles are low-cost fillers and are naturally abundant. They can also easily absorb organic molecules like chlorhexidine (CHX) and farnesol or be modified with silver, fluoride ions, and QPAIs—via eco-friendly, cost-effective, and direct ion exchange reactions—to produce dental composite restoratives exhibiting exceptional antimicrobial [25,26,27,28] and mechanical properties [29,30,31]. In terms of clay content, various experimental light-cured Bis-GMA/triethylene glycol dimethacrylate (TEGDMA)-based resin systems with individual clay fillers were studied with promising results, either with low (up to 20 wt%) [25,28,29,32,33,34,35,36] or high clay loadings (up to 65 wt%) [37,38,39,40,41]. To ensure high ultimate mechanical strength, other studies have reported the development of experimental dimethacrylate-based nanocomposite resins reinforced with clay nanoparticles and glass microfillers [26,42,43,44,45] or silica nanofillers [46,47] with a total inorganic filler content of 50–75 wt%.

Most of the above experimental models of dental nanocomposite resins meet the mechanical and physicochemical requirements imposed on conventional restoratives; however, they cannot be considered ready-to-use dental biomaterials because the Bis-GMA/TEGDMA volume ratio and filler content do not always match those of commercial formulations. Moreover, the suggested dental resin compositions lack specific additives, such as dyes for color adjustment, UV stabilizers for color stability, and inhibitors, that prevent premature polymerization during the production process, long storage, and clinical handling. From this perspective, it is important to consider these chemical factors and their possible interactions when developing new dental composites by incorporating clay nanoparticles. To the best of our knowledge, there is limited literature meeting such a crucial condition. Elmergawy et al. [48] evaluated the physical and mechanical properties of dental composites after the incorporation of 0.5–1.5 wt% PMMA-modified MMT and Cloisite 20A hydrophibic clay nanoparticles into the composition of a commercial flowable nanohybrid resin composite, presenting interesting results related to their mechanical behavior. Such low-viscosity resins are mostly recommended for anterior tooth restorations and filling small class I cavities, but they are not designed for the rapid, single-increment filling of large posterior restorations. However, no experimental data are currently available regarding the insertion of low amounts of rather hydrophilic organoclays into high-viscosity dental restoratives, which inherently contain higher filler content than flowable composites and are used for both anterior and posterior tooth occlusal surface restorations. Although a recent study reported the utilization of 0.25 wt% Cloisite 30B nanoclay with hydroxy content in PMMA-based acrylic dental resins, the latter are limited to the fabrication of dental occlusal devices and not recommended for direct tooth restorations as the dimethacrylate-based dental composite resins are [49]. Consequently, further research is warranted to address these unexplored areas and bridge the gap in the existing literature.

The aim of this study was to investigate the effect of the incorporation of an organically modified nanoclay containing hydroxyethyl groups on the morphological, mechanical, and physicochemical properties of a high-viscosity commercially available dental restorative material. Gaining a clearer understanding of these interactions and their impact on material performance is expected to provide insights for optimizing established dental composite formulations and supporting the development of restorative materials that better meet contemporary clinical requirements.

## 2. Materials and Methods

### 2.1. Preparation of the Unset Dental Composite Pastes

Suitable amounts (0.5, 1, 3, and 5 wt%) of clay nanopowder (Nanomer^®^ I.34MN, Nanocor Company, Hoffman Estates, IL, USA) were gradually incorporated into a microhybrid composite resin (Spectrum^®^ Universal Microhybrid Composite Restorative Dentsply DeTrey GmbH, Konstanz, Germany) using a hand spatulation method at ambient temperature under dark conditions. The resulting pastes were further mixed in a mortar and pestle until homogeneous blends were achieved and then ultrasonicated for 10 min. The specific composition of the starting materials is provided in Table 1, while the dental composite resins prepared at different clay concentrations are detailed in Table 2.

### 2.2. Instrumentation

Scanning electron microscopy (SEM) was conducted using a field emission scanning electron microscope, JEOL JSM-6390LV (JEOL USA, Inc., Peabody, MA, USA), equipped with an INCA X-act energy dispersive X-ray spectroscopy (EDS) microanalytical system (Oxford Instruments plc, Abingdon, Oxford, UK) operating at a 20 kV accelerating voltage and a magnification of ×1000.

X-ray diffraction spectroscopy (XRD) measurements were performed using a MiniFlex II XRD system (Rigaku Co, Tokyo, Japan) with CuKα radiation (λ = 0.154 nm) in the angle 2θ range from 2° to 10° at steps of 0.05° with a counting time of 5 s per step. All the dental composite samples were in the form of cured films. Clay nanopowder was also scanned for comparison reasons.

Attenuated total reflection–Fourier transform infrared (ATR–FTIR) spectra acquisition and photopolymerization kinetics were performed by placing a small amount of each composite between two translucent Mylar strips, which were pressed to produce a very thin film. The films of unpolymerized composites were exposed to visible light using a light-emitting diode (LED) polymerization unit (Bluephase^®^ Style M8, Ivoclar Vivadent AG, FL-9494, Schaan, Liechtenstein, Austria) at 800 mW/cm^2^ ± 10%, whose output irradiance was checked by a radiometer (Hilux, Benlioglu Dental Inc., Ankara, Turkey). After light irradiation, the films were immediately scanned using an ATR–FTIR spectrometer (Cary 630, Agilent Technologies, Mulgrave, Victoria 3170, Australia) with MicroLab software (version 4.6) at different curing times (0, 3, 5, 10, 15, 20, 30 s). Each sample was scanned in the range of 4000–600 cm^−1^. The resolution of the equipment was 4 cm^−1^ and the number of scans was 32. The areas of the aliphatic C=C peak absorption at 1637 cm^−1^ and the aromatic C=C peak absorption at 1608 cm^−1^ were determined utilizing a baseline technique that proved the best fit to the Beer–Lambert law [50]. The aromatic C=C vibration was used as an internal standard. The percentage degree of monomer conversion (DC %) of the cured specimen, which indicates the percentage of double carbon bonds reacted at each time period, was determined according to the equation (*n* = 5):

(1) $$DC\left(\%\right)=\left[1-\frac{{\left(\frac{A_{1637}}{A_{1608}}\right)}_{polymer}}{{\left(\frac{A_{1637}}{A_{1608}}\right)}_{monomer}}\right]\times 100$$

Disk-shaped specimens were created by filling a Teflon mold (15 mm × 1 mm) with composite pastes, then covering the mold with Mylar strips on both sides. A UV–Vis spectrophotometer (UV-2401 PC, Shimadzu, Tokyo, Japan) was used to record the light transmittance spectra in the wavelength range of 330–800 nm, employing an integrating sphere sample module. A baseline measurement was first taken with an unfilled mold placed between two Mylar strips. Subsequently, the light-transmission analysis was performed on the unset resin composite pastes.

Polymerization shrinkage was measured using the “bonded-disk” method, initially published and further refined by Watts and colleagues [51,52,53]. Briefly, a disk-shaped, unset specimen measuring 1.0 × 8.0 mm was prepared and centrally placed on a 3 mm-thick rigid glass plate. A flexible cover-slip diaphragm, supported by an outer brass ring with an internal diameter of approximately 15 mm, was rested on the top of the specimen to be adherent. A uniaxial linear variable displacement transducer (LVDT) measuring system was positioned centrally on the cover slip. The LVDT signal was transmitted to a computer via a transducer indicator (E 309, RDP Electronics Ltd., Wolverhampton, UK), and a high-resolution analog-to-digital converter (ADAM-4016 acquisition module) was used alongside datalogger software (Advantech Adam/Apax.NET Utility, version 2.05.11). Measurement data were recorded by continuously irradiating the specimens with the previously described LED light-curing unit for 5 min, directly underneath the glass plate at room temperature. Five repetitions (*n* = 5) were performed for each specimen. Strain was calculated as:

(2) $$\varepsilon (\%)=100\times \frac{\Delta L}{L_{0}}$$

where ε(%) represents the strain (%), and ΔL and L_0_ are the shrinkage displacement and the initial specimen thickness, respectively.

The determination of water sorption and solubility parameters was conducted following ISO 4049 [54]. A Teflon mold was filled with each unset dental composite to produce disk-shaped specimens (15 mm × 1 mm). The specimens (*n* = 5) were cured using the specified LED curing unit by irradiating for 40 s on each side, targeting nine preselected overlapping irradiation areas. Environmental conditions were maintained constant (22 °C, 40% relative humidity, 1 atmosphere) during the photocuring process. The disks were stored in a desiccator and then preconditioned at 37 ± 1 °C for 24 h. Afterwards, the specimens were transferred to another desiccator (23 ± 1 °C) and kept there for 2 h. They were then weighed (±0.1 mg accuracy) using an analytical balance (Sartorius TE 124S, Sartorius AG, Göttingen, Germany). This procedure was repeated until the materials reached a constant mass (*m*_1_). Their volume (*V*) was calculated based on their mean thickness and diameter, measured with a digital micrometer (±0.02 mm accuracy). Subsequently, the specimens were stored in water at 37 ± 1 °C. After one week, they were removed, dried, and weighed (*m*_2_). The disks were then placed back into the desiccator until a constant mass (*m*_3_) was achieved. The water sorption parameter, *W_sp_* (μg/mm^3^), was calculated using the formula:

(3) $$W_{sp}=\frac{m_{2}-m_{3}}{V}$$

where *m*_2_ is the mass of the disc (μg) after storage in water for 1 week, *m*_3_ is the mass of the reconditioned disc (μg), and *V* is the volume of the disk (mm^3^).

The solubility parameter, *W_sl_* (μg/mm^3^), was calculated according to the following equation:

(4) $$W_{sl}=\frac{m_{1}-m_{3}}{V}$$

where *m*_1_ is the conditioned mass (μg) of the disk before the storage in water.

For flexural tests, bar specimens were prepared by filling a Teflon mold (2 mm × 2 mm × 25 mm) with unpolymerized paste, following ISO 4049 [54]. The mold surfaces were overlaid with glass slides covered with a Mylar sheet to avoid air entrapment and adhesion of the final set material. The assembly was held together with spring clips and irradiated by overlapping on both sides, as described above. Each overlap irradiation lasted for 20 s (as recommended by the manufacturer). Ten specimen bars (*n* = 10) were prepared for each composite. The specimens were stored in water at 37 °C for 1 week after curing. Afterwards, they were bent in a three-point transverse testing rig with 20 mm between the two supports (three-point bending). The rig was fitted to a universal testing machine (Testometric AX, M350-10kN, Testometric Co. Ltd., Rochdale, UK). All bend tests were carried out at a cross-head speed of 0.5 mm·min^−1^ until fracture occurred. The load and the corresponding deflection were recorded. The data were collected and processed using the WinTestAnalysis CX Version 3.5.30.10 software. The flexural modulus (E) in GPa and flexural strength (σ) in MPa were calculated according to the following equations:

(5) $$E=\frac{F_{1}l^{3}}{4bd_{1}h^{3}}10^{-3}\;and\;\sigma =\frac{3F_{max}l}{2bh^{2}}$$

where F_1_ is the load recorded in N, F_max_ is the maximum load recorded before fracture in N, l is the span between the supports (20 mm), b is the width of the specimen in mm, h is the height of the specimen in mm, and d_1_ is the deflection (in mm) corresponding to the load F_1_.

### 2.3. Statistical Analysis

Descriptive statistics were calculated using the mean values of element content (wt%), degree of conversion (DC%), strain (%), water sorption (μm/mm^3^), solubility (μm/mm^3^), flexural modulus (GPa), flexural strength (MPa), and standard deviation (SD). The assumption of normality was assessed for the variables using the Shapiro–Wilk test, followed by the Levene test for homogeneity of variances. One-way analysis of variance (ANOVA), followed by a post hoc Tukey test for multiple-testing adjustment, was used to evaluate the association of DC (%) with nanoclay filler loading, while the DC (%) comparisons between different tested light-curing time processes were based on *t*-tests for equality of means. Non-parametric Kruskal–Wallis tests, adjusted by Bonferroni corrections for multiple comparisons, were applied to determine the correlation of element content (wt%), strain (%), water sorption (μm/mm^3^) and solubility (μm/mm^3^), flexural modulus (GPa), and strength (MPa) with nanoclay filler loading. The strain (%) comparisons between various light-curing times were based on *t*-tests for equality of means. Statistical analysis was performed using SPSS Statistics (V.28, IBM, Armonk, NY, USA). The statistical significance level was set at *p*-value ≤ 0.05.

## 3. Results and Discussion

In the current research work, new dental composite resins were synthesized by incorporating organoclay nanoparticles containing hydroxyethyl groups into an integral formulation of a commercial high-viscosity dental composite resin. To facilitate hand spatulation of the prepared composite pastes, as suggested by other researchers [36,38,40,48], the clay nanofiller loading was limited to 0.5–5 wt%, ensuring the paste handling properties closely resembled those of the pristine commercial microcomposite. According to the data in Table 1, the base monomer in dental composite resin’s formulation is a urethane modified Bis-GMA resin. The latter constitutes a Bis-GMA variant where hydroxy groups are replaced by urethane groups (HN–(C=O)–O) to produce a monomer of lower viscosity and water uptake [55]. Although the manufacturer does not refer to the incorporation of TEGDMA as a reactive diluent, such urethane-modified Bis-GMA resins have been reported to form cross-linked networks when combined with TEGDMA [56]. Herein, the visible light photoinitiator system contributing to the initiation of free radical polymerization consists of the camphorquinone/ethyl-4(dimethylamino)benzoate (CQ/DMAEMA) redox system, which is typically used in light-cured dental composite resins. Taking account of the above information, it can be stated that upon light irradiation, the active monomers react via their vinyl groups, resulting in a highly cross-linked polymer network, which is responsible for the ultimate solidification of the resin.

### 3.1. Structural and Morphological Characterization

**Scheme 1:**
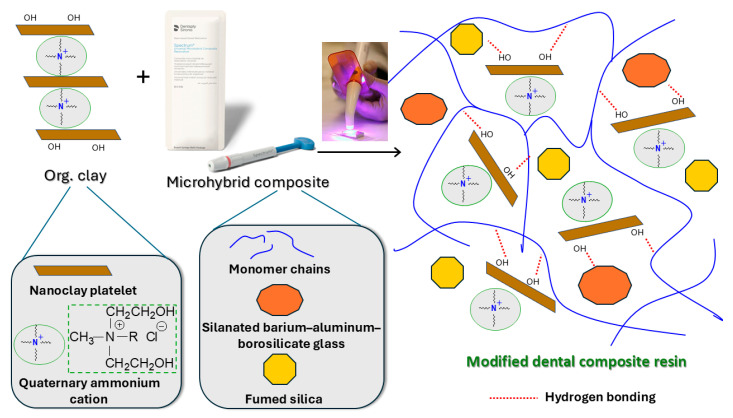
A proposed representation of nanoclay particles’ direct incorporation into the polymer network of a commercially available light-cured dental microhybrid composite resin.

**Figure 1:**
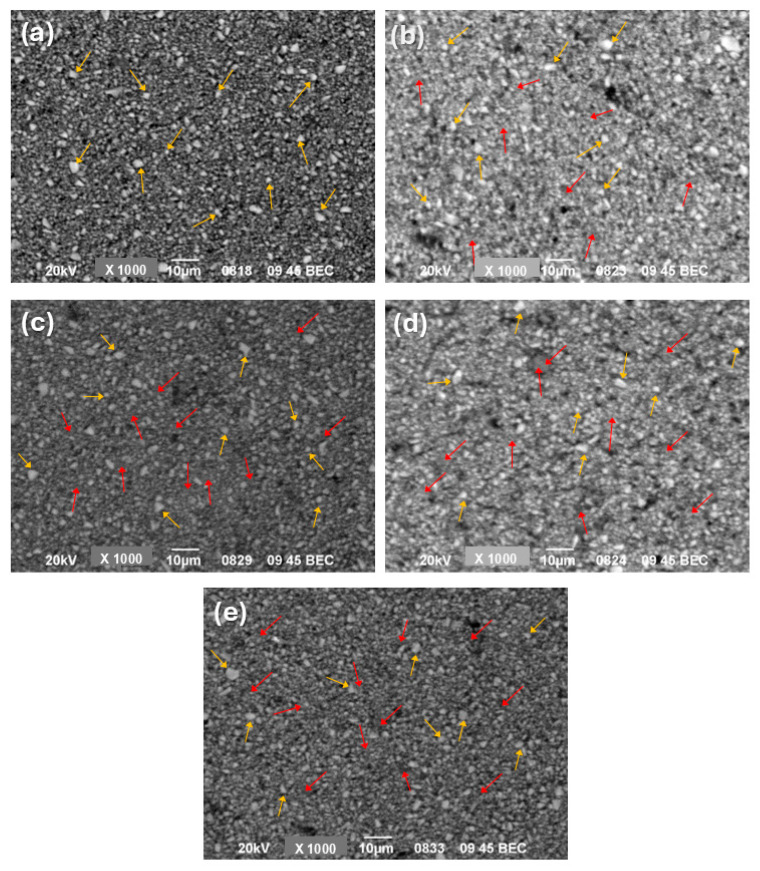
SEM images captured for (**a**) conventional dental composite resin (DCR0) and synthesized DCRs containing (**b**) 0.5 wt% (DCR0.5), (**c**) 1 wt% (DCR1), (**d**) 3 wt% (DCR3), and (**e**) 5 wt% (DCR5) organoclay nanoparticles. Yellow arrows indicate larger inherent fillers of DCR, while red arrows indicate the regions containing clay nanoparticles.

SEM images of the pristine microhybrid composite (DCR0, Figure 1a) disclosed distinct bright dots corresponding to larger inherent inorganic fillers (yellow arrows) surrounded by smaller particles (grey sections) distributed within the polymer matrix (narrow dark areas). Microhybrid composites usually contain 66–80 wt% fillers [57,58,59]. Hence, the organic matrix areas were expected to be more restricted compared to non-organic ones. The boundaries of the bright dots were no longer clearly detected by the gradual insertion of clay nanoparticles (Figure 1b–e), as individual clay platelets may enter the free space (red arrows) between the pre-existing larger and smaller particles. In addition, the organic matrix areas of modified composites appeared to increase despite a decrease in organic content as the clay mass fraction increased. A possible explanation could be that the incorporation of clay nanofillers might favor hydrogen bonding between the hydroxy groups of clay organomodifier and glass–silica particles of the dental composite (Scheme 1), thus affecting the distribution of inorganic particles into the existing polymer matrix and allowing for broader organic regions and possible disruption of normal packing density. Chemical interactions induced by Cloisite^®^ 30B nanoclay (containing hydroxy groups) were previously reported by other researchers [38].

**Figure 2:**
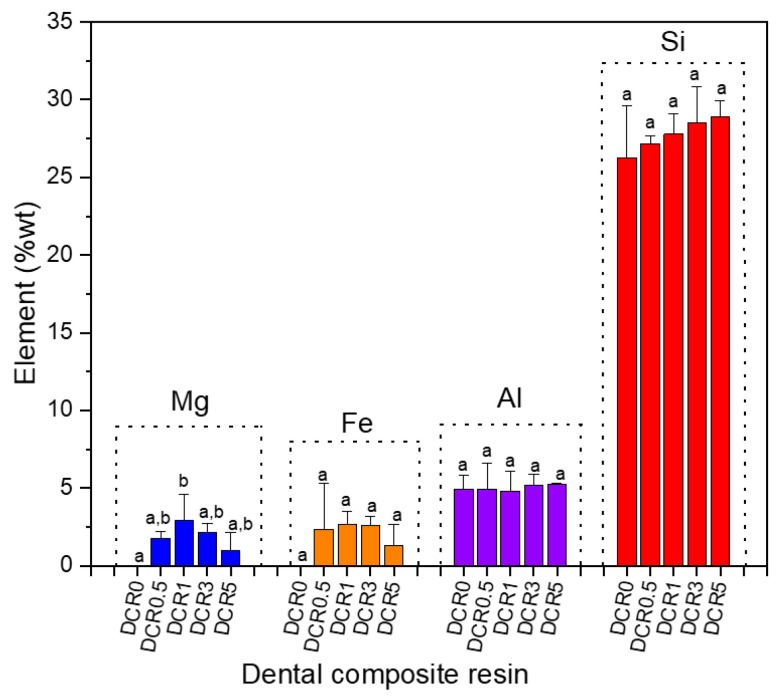
Element weight percentage (median values ± interquartile range (IQR)) (*n* = 4) determined by means of EDS analysis for the synthesized dental composite resins (DCRs) at different levels of clay nanoparticles (0–5 wt%). The same lowercase letters on the bars indicate no statistically significant difference within each element group (*p* < 0.05).

The experimental data obtained through EDS analysis are shown in Figure 2. The variations in aluminum and silicon levels were not statistically significant across the studied composites (*p* < 0.05). All DCRs having clay nanoparticles showed higher amounts of silicon than DCR0, thus ensuring the successful incorporation of clay nanoparticles. A small increase in the aluminum amount was determined for composites having 3 and 5 wt% organoclay (DCR3 and DCR5), respectively. Saridou et al. also mentioned enhanced amounts of aluminum when organomodified clay was gradually added to Bis-GMA/TEGDMA formulations [47]. Moreover, the dental composites exhibited measurable amounts of magnesium and iron, which were not detected on the surface of the pristine composite (DCR0). Since these elements likely originate from clay content, this finding could be linked to the surface presence of nanoclay, which became more evident when 1–3 wt% clay nanoparticles were used. As expected, there were statistically significant differences in magnesium content between DCR0 and DCR1 composites (*p* < 0.003). Overall, the maximum magnesium and iron levels were found for the DCR reinforced with 1 wt% organoclay nanoparticles.

**Figure 3:**
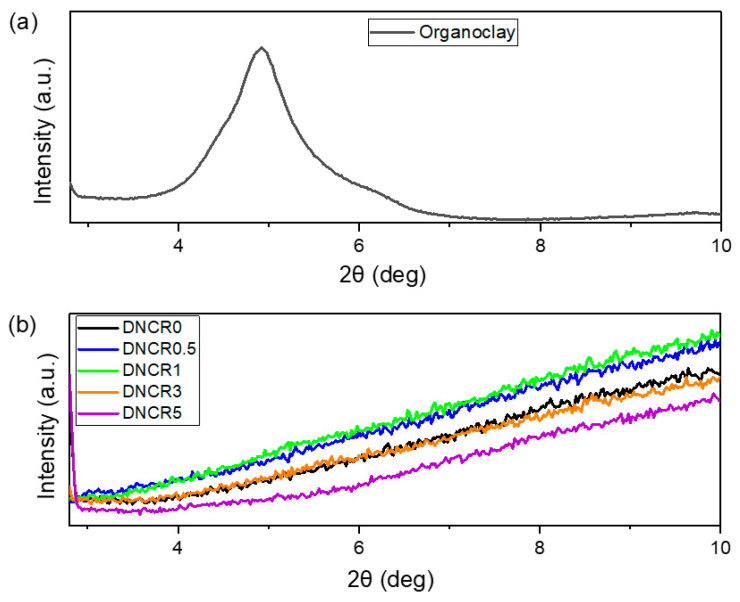
XRD patterns of (**a**) organoclay nanoparticles, (**b**) dental composite resin (DCR0), and synthesized composite resins containing different nanoclay filler mass fractions (DCR0.5-DCR5).

The XRD diffractograms of the organoclay nanopowder, pristine dental composite resin, and developed composites are illustrated in Figure 3. According to the spectrum acquired for pure nanoclay (Figure 3a), a strong diffraction peak was observed at approximately 2θ = 4.9^ο^. However, no (001) plane peaks were found for the synthesized composites (Figure 3b). The lack of crystalline clay in the blank DCR0 could justify the absence of the aforementioned peak. Regarding DCRs reinforced with clay nanoparticles (DCR0.5-DCR5), no more (001) diffraction peaks of crystalline clay were visible, possibly due to extensive clay layer separation, which disrupts the clay layer stacking in the crystal lattice. Too large spacing between layers or disorientation of them may suggest exfoliated clay conformations [60,61,62]. During exfoliation, the developed macromolecular chains penetrate the clay galleries, leading to structural delamination and random dispersion of individual mineral layers into the polymer network (Scheme 1). Moreover, in such low amounts of clay, the exfoliated conformations may be enhanced by the two hydroxy groups presented in the modifier structure. These could interact with the polar groups of the resin’s monomers and promote high compatibility between the organic matrix and dispersed nanofillers [8]. Regarding Bis-GMA/TEGDMA-based experimental systems, Mahmoodian et al. reported clay exfoliation state for composites containing 1 wt% Cloisite^®^ 30B nanoclay [36], while Menezes et al. also proved exfoliated structures when organoclay loading of 2.5 wt% was dispersed in Bis-GMA/UDMA/TEGDMA-based systems [43]. On the other hand, higher Cloisite^®^ 30B loadings (50–65 wt%) applied in a Bis-GMA/TEGDMA matrix by Campos et al. resulted in both exfoliation and intercalation phenomena [38].

**Figure 4:**
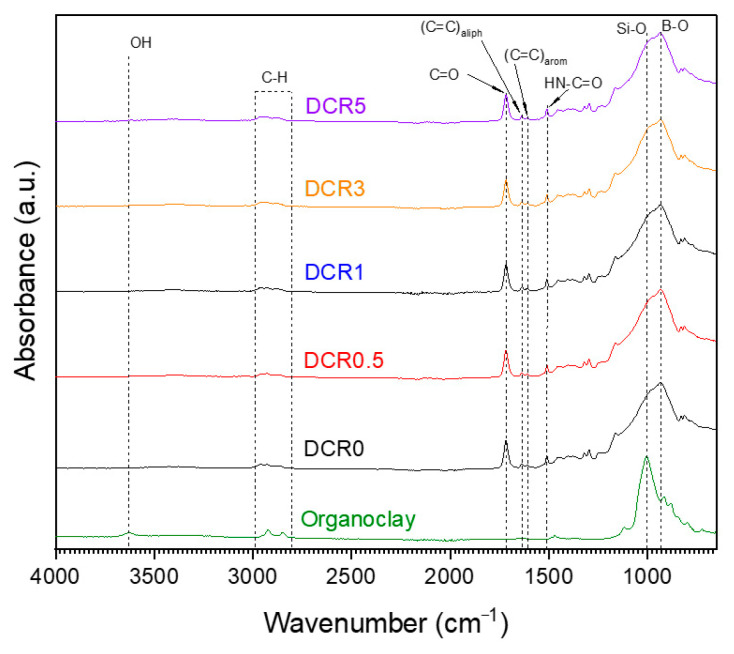
ATR–FTIR measurements taken for organoclay nanoparticles, commercially available dental composite resin (DCR0), and the synthesized dental composite resins (DCR0.5-DCR5).

According to Figure 4, the characteristic peak assignments of the acquired ATR–FTIR data are as follows: 3630 cm^−1^ OH stretching vibrations (organoclay), 2953–2853 cm^−1^ symmetric and asymmetric stretch of methylene (-C-H) groups (organoclay and composites), 1714 cm^−1^ C=O stretch due to ester groups (composites), 1636 cm^−1^ C=C aliphatic stretch of vinyl groups (composites), 1608 cm^−1^ C=C aromatic stretch (composites), 1510 cm^−1^ H-N-C=O amide II (composites), 1318 cm^−1^ -CH- bend (composites), 1292 cm^−1^ C–O bend (composites), 1000 cm^−1^ Si–O stretch (organoclay and composites), and 935 cm^−1^ B–O stretch (composites). Since no additional peaks were detected in modified dental composites, it can be concluded that no covalent bond alterations took place by inserting clay nanoparticles.

### 3.2. Evaluation of Light-Transmittance

**Figure 5:**
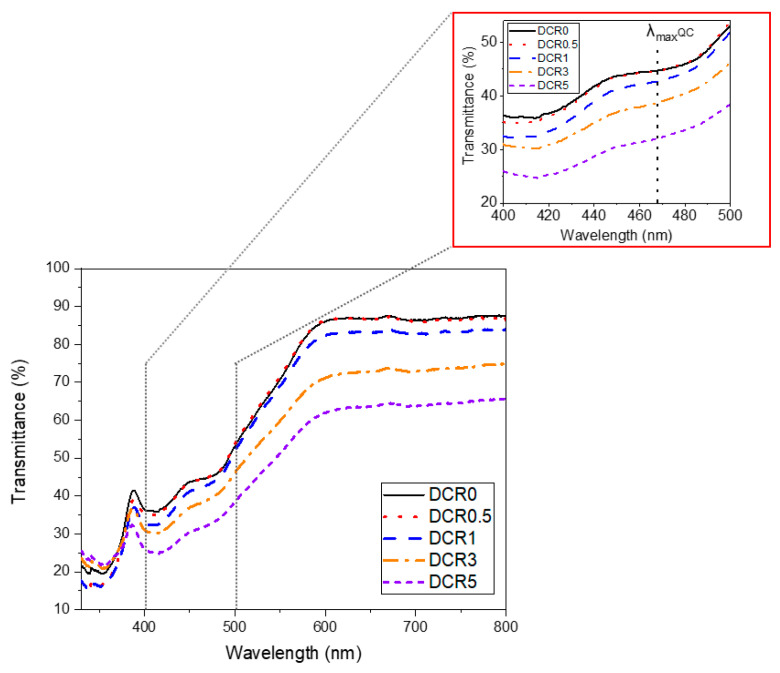
Full light transmittance spectra of the conventional dental composite and the synthesized dental composite resins. A magnification of the plots in the range of 400–500 nm includes the λmaxQC (468 nm), which corresponds to the maximum absorbance wavelength of the photoinitiator camphorquinone.

Representative full UV–Vis spectra obtained for the synthesized DCRs are shown in Figure 5. Additional magnified plots include the light transmittance response over the wavelength range of the light-curing lamp (430–490 nm). The wavelength corresponding to the maximum absorbance of camphorquinone (λ_max_ = 468 nm) is also marked. It is evident that increasing the number of clay nanoparticles gradually decreased the transmitted light intensity. This effect was more noticeable, as expected for mass fractions above 1 wt%. Furthermore, the light transmittance was extremely attenuated for the composite having 5 wt% nanoclay. Refractive index differences between inorganic fillers and the organic matrix, as well as factors such as monomer type, filler type, filler content, and filler particle shape, influence the optical properties of dental composite resins [63,64]. Since the other components of the commercial dental composite remain unchanged, the observed reduction in the modified composites’ transparency to light, along with the XRD and SEM results, suggests that light absorption and scattering are caused by the presence of clay. This phenomenon was mainly attributed to the abundance of exfoliated clay platelets at higher nanofiller amounts, as the random orientation of the clay particles can alter the direction of the incoming light beams and ultimately increase light diffusion. A similar trend was reported by Munhoz et al. when organically modified clay was incorporated into urethane dimethacrylate (UDMA)/Bis-GMA/TEGDMA-based experimental systems at nanoclay contents up to 7.5 wt% [31].

### 3.3. Assessment of the Degree of Conversion (DC)

**Figure 6:**
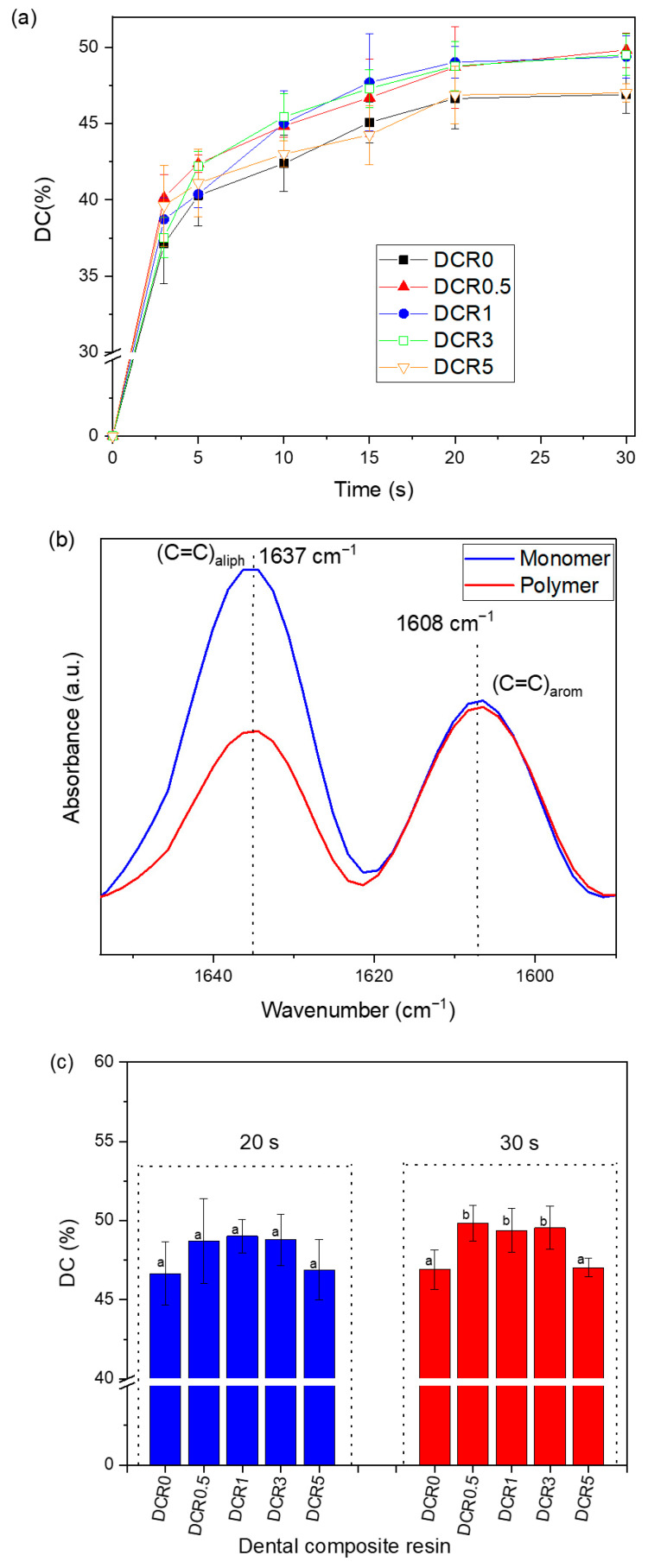

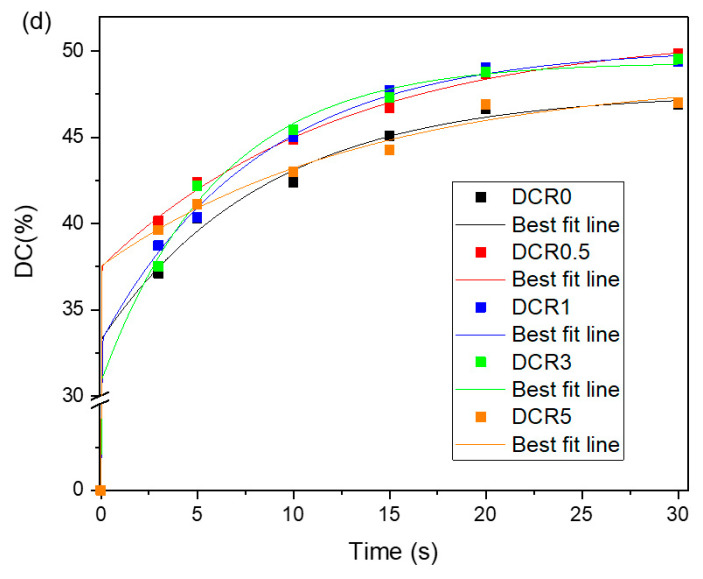
(**a**) Degree of conversion versus light-curing time for the conventional dental composite and resins synthesized with varying amounts of clay nanofillers. (**b**) ATR–FTIR spectra with measured peak areas (1636 and 1608 cm^−1^) used to calculate the percentage degree of conversion (DC %) for uncured and cured DCRs. (**c**) DC% (mean values ± standard deviation) measured for dental composite resins after 20 s and 30 s of light-curing (*n* = 5). Different lowercase letters on the bars indicate statistically significant differences within each curing time group (*p* < 0.05). (**d**) Representative DC time experimental data and lines of best fit through all data points corresponding to the two exponential approximate functions.

The monitoring data for the degree of conversion over irradiation time are plotted in Figure 6a, while Figure 6b clearly presents the FTIR absorbance peaks of unset and set DCRs selected for the calculation of DC values. The final DC values after 20 s and 30 s of light irradiation are shown in Figure 6c. All composites exhibited final DC values ranging from 46.66% to 49.83%. These results fall within the range of 40.96–49.18%, as reported by Catelan et al., for a microhybrid composite (4 Seasons, Ivoclar Vivadent) irradiated for 20 s with a LED unit of 700 mW/cm^2^ irradiance [65]. Costa et al. also described a microhybrid resin composite (Gradia Direct-GC) yielding 43.06–44.72% degree of conversion after 20 s irradiation with a light-curing unit of 1000 mW/cm^2^ [66]. Ucuncu et al. mentioned DC values within 46.86–60.01% for the Myra microhybrid (Dentac) after 20 s irradiance with a LED unit ≥800 mW/cm^2^ [67]. According to Figure 6a, an abrupt increase in reaction rate occurred during the initial 3 s, which corresponded to DC = 37.5–40%, perhaps due to a gel effect associated with diffusion-controlled phenomena [68]. This auto-acceleration effect was more intensive for the modified composites (DCR0.5-DCR5) than the conventional one (DCR0), as the co-existence of clay platelets with the inherent glass particles and the formed cross-linked network could further reduce the free volume of the reactant dimethacrylate monomers. This effect may hinder the diffusion of the developed macro-radicals and their ability to locate and react with each other to terminate the reaction. Consequently, the radical concentration may increase locally, resulting in the enhancement of the polymerization rate. After 3 s of light irradiation, the DC% increased gradually because the reduction in the termination reaction rate was less steep. The relatively high DC% values achieved for modified composites within the 5–20 s time interval were still maintained by the presence of nanoclay particles. Finally, a plateau of the kinetics curves was recorded within 20–30 s of light-curing, as the reaction rate tends asymptotically to zero and the polymerization terminates at DC values lower than 50% due to the established glassy state of the remaining monomer/polymer mixture [68].

The influence of clay filler loading on the ultimate DC values is also shown in Figure 6c. Regardless of the light-curing mode, the addition of nanoclay improved the final conversion. No statistically significant differences in DC% were observed within the 20 s light-curing mode groups. When the 30 s curing mode was selected, statistically significant differences were observed for DCR0 resin compared to DCR0.5 (*p* = 0.007), DCR1 (*p* = 0.025), and DCR3 (*p* = 0.016). Significant differences were also detected for DCR5 against DCR0.5 (*p* = 0.009), DCR1 (*p* = 0.035), and DCR3 (*p* = 0.023). Considering 20 s light irradiation, the maximum DC% (49.03%) was achieved by using 1 wt% clay, while curing (49.83%) by adding 0.5 wt% nanofiller for 30 s (Figure 6c). The obtained results revealed that the usage of 0.5–3 wt% nanoclay enhanced the DC% of the dental composite resins. At relatively higher clay content regimes (3–5 wt%), more exfoliated opaque layers could prevent light penetration, as previously mentioned, thereby limiting the augmentation rate of DC%. At such low amounts of nanoclay, decreased values of DC% upon the gradual increment of clay content were reported by Mahmoodian et al. [36], Elmergawy et al. [48], and Menezes et al. [42,43], while DC fluctuations were presented by Avila et al. [33]. On the other hand, Degrazia et al. [35] found higher DCs for nanocomposites having up to 10 wt% halloysite clays, while Boaro et al. [34] did not detect any DC changes when CHX clay was added to dental composites. Nonetheless, the augmented final DC% of the current modified composites could also be facilitated by a secondary mechanism that supplements the aforementioned gel effect. In particular, the reactive triplet state of camphorquinone (CQ*), originating from the CQ excited single state due to visible light irradiation, might abstract hydrogen atoms from hydroxy groups attached to the nanoclay organic modifier (HO-CH_2_CH_2_-N^+^-) and to the edges of clay platelets (Al–O–H groups) (Scheme 1) [69,70]. In this manner, clay nanoparticles could be converted into free-radical vehicles, which are highly reactive toward monomer double bonds. Consequently, both the suggested auto-acceleration effect and hydrogen abstraction mechanism could overlap the effect of attenuated light penetration and contribute to the final DC increment.

The irradiation time proposed by the manufacturer was 20 s even though 30 s light-curing is often applied for dental composites in clinical practice to ensure complete polymerization of the restorative material. The above conditions were a rationale for studying DC% at irradiation times longer than 20 s. The data in Table 3 showed no statistically significant differences when comparing the curing time modes (20 s vs. 30 s) at the same clay nanofiller percentage (*p* < 0.005). Al-Qahatani et al. also confirmed no statistically significant differences of DC% for the microhybrid Spectrum TPH cured at 20 s and 40 s, respectively [71]. Overall, it can be concluded that the optimal DC (49.83%) may be accomplished by irradiating the composites for 30 s in the presence of 1 wt% nanoclay.

A five-parameter model described by the superposition of two exponential functions was also used to best fit the experimental data of DC against polymerization time:

*y* = *y*_0_ + *a* (1 − e^−*bx*^) + *c* (1 − e^−*dx*^) (6)

where *y* corresponds to the DC (%); *y*_0_ is the y-intercept; and *a*, *b*, *c*, and *d* are modulation factors used to describe the polymerization kinetics during the gel phase (*a* and *b*) and the glass phase (*c* and *d*). The calculated DC values of all composites were plotted again versus time, and a line of best fit was fitted through the measured points (Figure 6d). Table 4 summarizes the calculated parameters *y*_0_, *a*, *b*, *c*, and *d*, as well as the coefficient determination R^2^ derived from the theoretical model. The *R*^2^ values revealed a satisfactory correlation between measured values and the approximate function. According to the best-fit parameters presented in Table 4, the estimated values of *y*_0_ are very small and approach zero in all cases, which is consistent with the expected physical behavior of the system. Based on Equation (6), when the time variable (*x*) is equal to zero, the response variable (*y*) becomes equal to *y*_0_. Considering that the degree of conversion (DC) should theoretically be zero at the initial time point (*x* = 0), the near-zero values obtained for *y*_0_ are therefore physically reasonable and support the validity of the fitting procedure. Assuming a long polymerization time (*x* → ∞), Equation (6) is converted to:

*y* = *y*_0_ + *a* + *c* (7)

The combination of fit parameters (Table 4) and Equation (7) enables the calculation of the theoretical ultimate DC (%). Comparisons between the theoretical results (Table 4) and ATR–FTIR data (Figure 6c) show that the suggested model produces DC (%) values that are close to the experimental data.

### 3.4. Study in Polymerization Shrinkage

**Figure 7:**
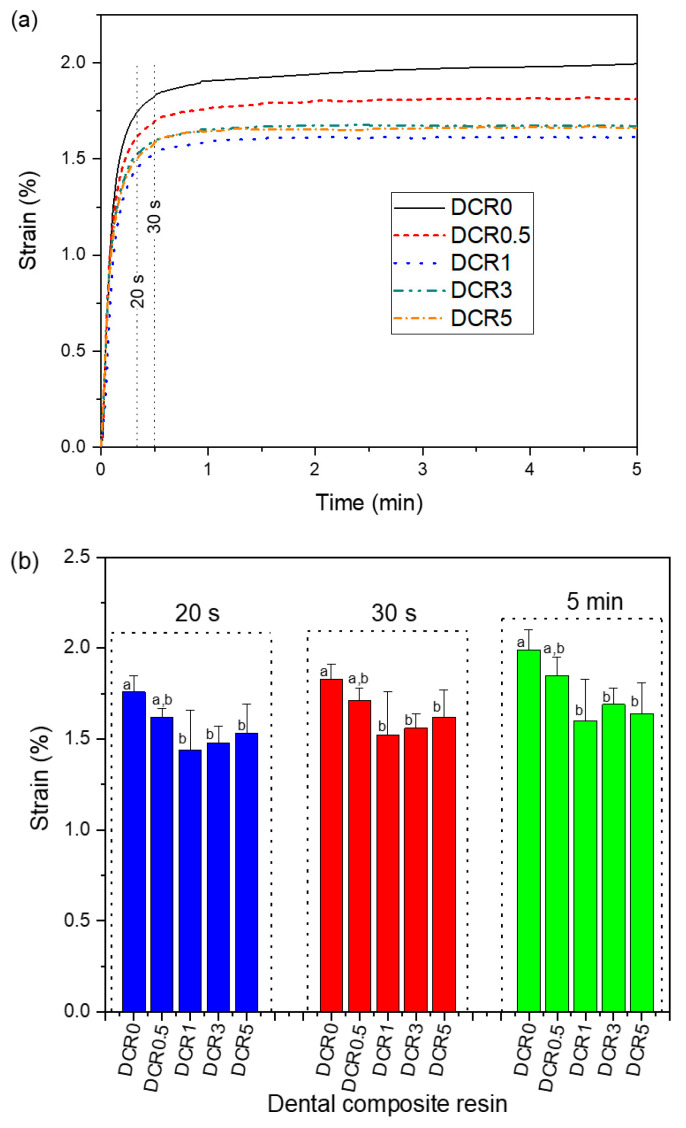
(**a**) Plots of shrinkage strain (%) against time during the polymerization process of DCRs containing 0–5 wt% nanoclay fillers. (**b**) Strain % (median values ± IQR) measured for dental composite resins after 20 s, 30 s, and 5 min of light curing (*n* = 5). Different lowercase letters on bars indicate statistically significant differences within each curing time group (*p* < 0.05).

Polymerization shrinkage–time curves for the synthesized dental composite resins are shown in Figure 7a, whereas the strain values (%) acquired at specific irradiation time intervals are shown in Figure 7b. Regardless of the irradiation time, all modified DCRs exhibited lower volumetric reduction through the polymerization process compared to the conventional composite. Previous studies suggested that the incorporation of 30–65 wt% Cloisite^®^ 20A and Cloisite^®^ 30B organoclays into the Bis-GMA/TEGDMA system reduced the polymerization shrinkage from 2.36 to 0.22% [38,39,40,41]. The strain values (%) fall within the above range after the usage of lower clay nanofiller contents. Consequently, these findings verified that the increment of the overall filler volume fraction through clay incorporation can further restrict the setting contraction by virtue of free volume reduction when monomer atoms move closer to form covalent bonds [72]. DCRs reinforced with 1–5 wt% clay nanoparticles showed statistically significant differences against DCR0 at every light-curing time (*p* < 0.05). It is evident that continuous irradiation over 20 s increases the induced shrinkage strain, regardless of the amount of clay filler, whereas in most cases statistically significant differences were detected within the intermediate time intervals (*p* < 0.05, Table 3). Furthermore, under each specific light-curing condition, the lowest setting contraction values were observed when 1 wt% of nanoclay was added. This finding suggests that such a small amount of 1 wt% clay is adequate to meet the clinical requirements for low polymerization shrinkage. With regard to clinical relevance, volumetric shrinkage due to developed polymerization stresses can result in marginal gaps between the composite and tooth enamel, favoring microleakage phenomena associated with secondary caries and restorative failure [73]. Ιt could be suggested that DCRs reinforced with 1 wt% clay nanofiller may ensure optimal performance in the 20 s light-curing mode. Resin matrix composition, filler content, and degree of conversion are the major factors influencing the volumetric shrinkage [72]. In terms of DCR1, it could be stated that the lower DC values after 20 s of light irradiation were contributory to the lower setting contraction in comparison to the 30 s curing mode.

### 3.5. Determination of Water Sorption and Solubility

**Figure 8:**
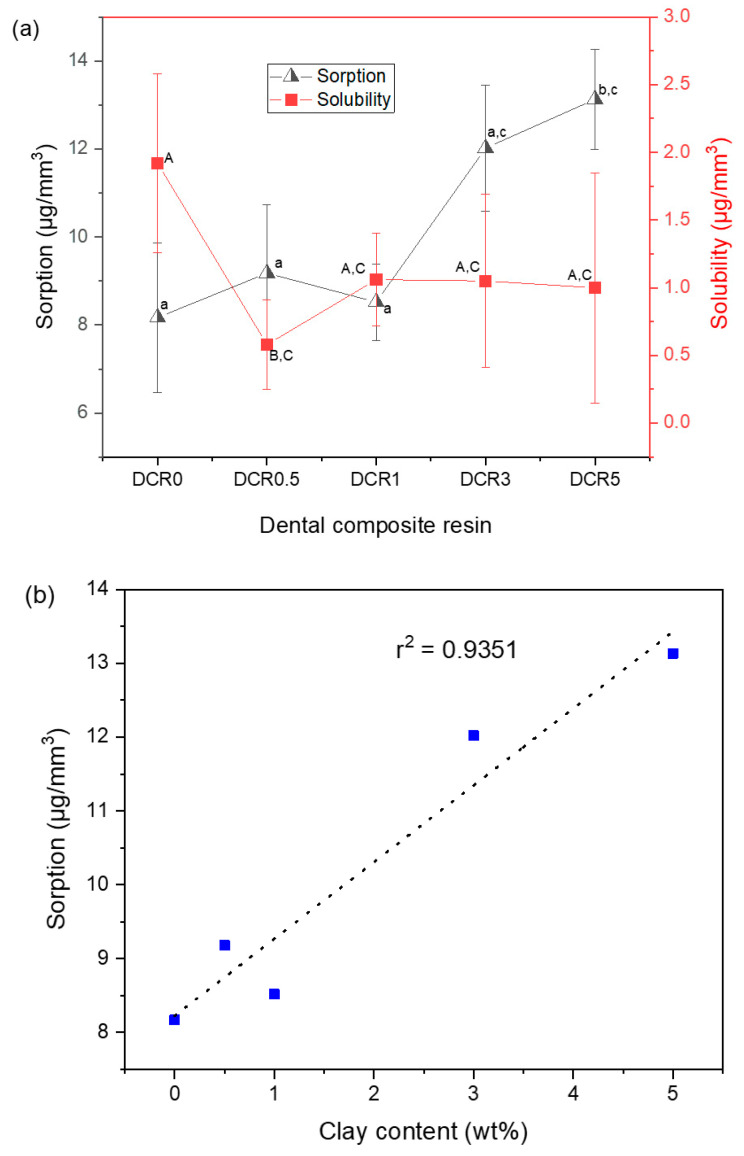
(**a**) Water sorption and solubility parameters (median values ± IQR) of DCRs enhanced with 0–5 wt% organoclay (*n* = 5). Different lowercase and uppercase letters indicate statistically significant differences between composite groups (*p* < 0.05). (**b**) Regression line of water sorption plotted against nanoclay filler mass fraction used for the synthesis of dental composite resins. The blue dots correspond to the experimental sorption values.

Figure 8a depicts the water sorption and solubility results gained for the studied composite materials. An increase in the sorption parameter was recorded upon the increase of clay nanofiller mass fraction. Specifically, the addition of 5 wt% nanoclay led to a statistically significant increase in water compared to the other composite groups (*p* < 0.05). No other significant differences were observed. According to Figure 8b, a linear correlation between sorption and the amount of clay was evident (r^2^ = 0.9351, y = 1.0397x + 8.2287). The deviation of the sorption values from the straight line upon increase of the organoclay content could be attributed to the disruption of normal packing density, as previously documented by SEM findings, which may render the dental composite heterogeneous with unpredictable water sorption behavior. In each case, the elevated sorption values could be attributed to the presence of hydroxyl groups attached to the quaternary ammonium cation clay modifier (Scheme 1), which may favor the hydrogen bonding with water molecules approaching the composite’s surface and finally contributing to sorption phenomena. The present results are consistent with previous studies reporting increased water sorption in dental composites when the nanoclay content is kept low [31,32,46]. Water accumulation can influence the matrix–filler bond stability, resulting in possible composite swelling, discoloration, low transparency, and the deterioration of mechanical performance [74,75]. However, the measurable sorption values did not exceed the upper limit specified by ISO 4049 for polymer-based restorative materials (<40 μg/mm^3^) [54]. Furthermore, the water sorption of DCR containing 1 wt% clay (8.52 μg/mm^3^) was similar to that of the conventional dental composite resin (8.17 μg/mm^3^). This observation denotes that among the different DCRs reinforced with nanoclay particles, the DCR1 was more favorable against water sorption. In the solubility tests conducted, it was found that all DCRs containing clay nanoparticles presented lower solubility than the conventional DCR, suggesting that the utilization of nanoclay in dental restorative materials is highly effective against resin solubility. Particularly, the DCR containing 0.5 wt% clay demonstrated statistically significant lower solubility (0.58 μg/mm^3^) than the conventional DCR (1.92 μg/mm^3^). All the studied composites met the solubility criterion per ISO 4049 (<7.5 μg/mm^3^) [54]. The relatively high DC values of modified composites when compared to the traditional composite resin (Figure 6) may be responsible for the resistance of modified DCRs to solubility. The literature data provide contradictory information in terms of the solubility changes owing to the presence of clay nanoparticles. Encalada-Alayola et al. [32] reported monotonically decreased solubility when the laminar Cloisite^®^ 30B clay was added at low levels into experimental Bis-GMA/tetraethylene glycol dimethacrylate (TTEGDMA)-based systems. On the other hand, Ritto et al. [46] found that increased solubility was directly correlated with elevated water sorption upon progressive reinforcement of dental composite with clay nanofillers, considering a two-way process involving water absorption followed by monomer leaching from the resin bulk. The assessment of the solubility parameter is crucial to predicting the long-term behavior and clinical performance of the dental restorative material [76]. Herein, the observed low solubility of modified DCRs may account for the limited leaching of unreacted monomers and contribute to their greater safety.

### 3.6. Determination of Mechanical Properties

**Figure 9:**
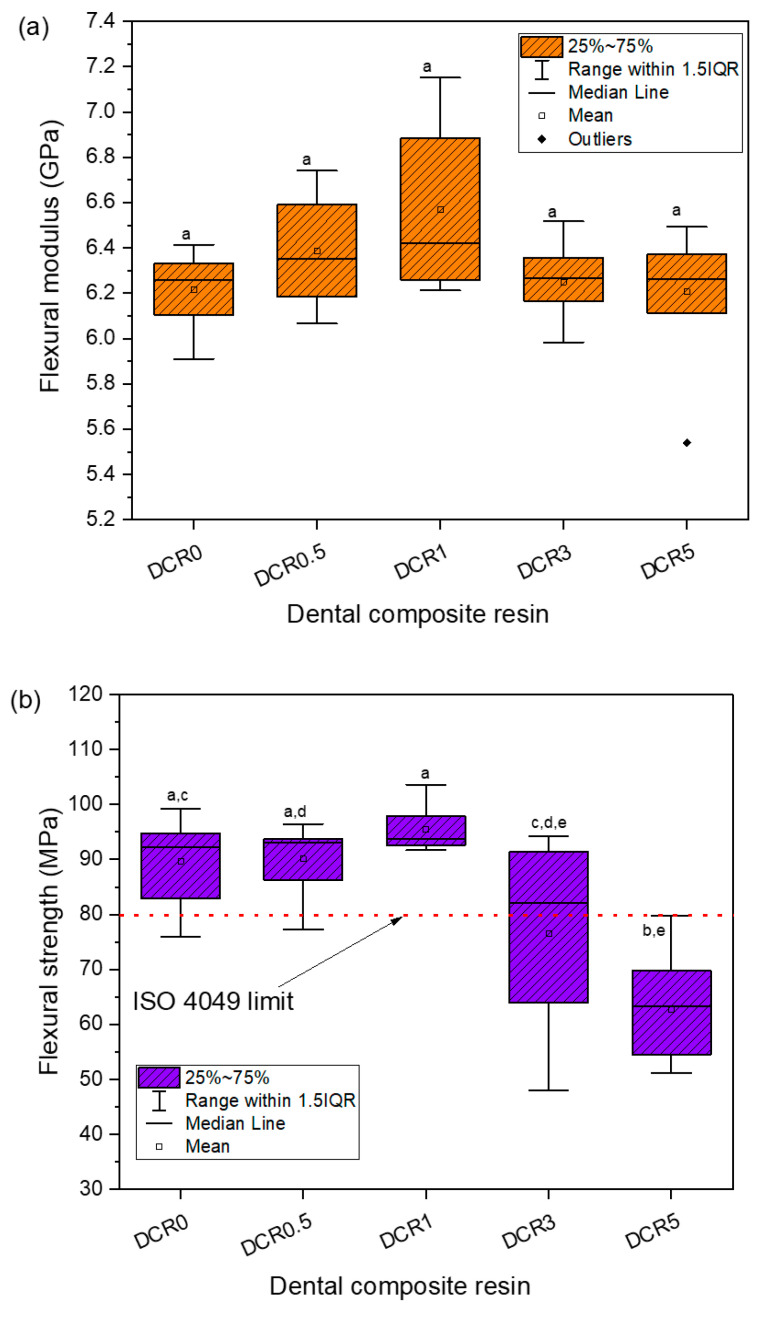

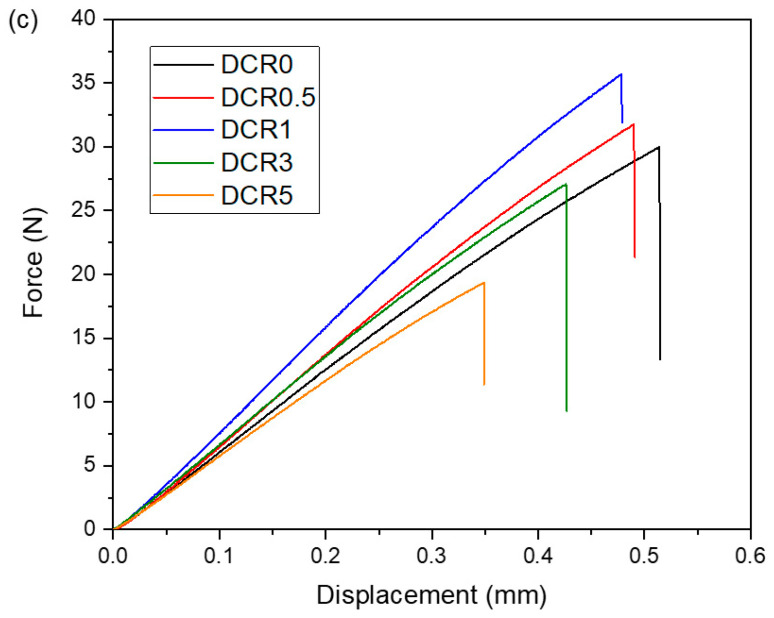
Box plots of (**a**) flexural modulus and (**b**) flexural strength determined for conventional and modified DCRs reinforced with 0–5 wt% organoclay nanofillers (*n* = 10). Different lowercase letters indicate statistically significant differences between composite groups (*p* < 0.05). (**c**) Representative graphs of applied force against the displacement for the studied dental composite resins.

The obtained flexural property data, representing the modulus and strength trends of the studied composites across the applied clay loadings, are illustrated in Figure 9a,b. The initial three-point bending records showing the applied forces versus the induced deformations of DCRs are presented in Figure 9c. Regarding the dental restoratives, elastic modulus is a measure of a material’s stiffness, indicating its resistance to non-permanent deformation provoked by loadings due to masticatory forces. It was found that incorporating clay nanoparticles into the polymer matrix might maintain the stiffness of the conventional microcomposite (*p* < 0.05). The maximum stiffness (6.56 GPa) was achieved by incorporating only 1 wt% nanoclay into the DCR, suggesting that the inherent rigidity of mineral clay combined with the enhanced DC may account for the increased stiffness. As the clay content reached 5 wt%, the flexural modulus was not further increased, probably due to the sorption phenomena dominating during DCR storage in water, which allowed liquid molecules to act as plasticizers, thus mitigating the yielded elastic modulus. The literature data have shown that the improvement in elastic modulus depends on the clay mass fraction. Encalada-Alayola et al. [32] reported a maximum modulus (around 1.4 GPa) for experimental Bis-GMA/TTEGDMA systems reinforced with 4 wt% Cloisite^®^ 30B clay, Menezes et al. [42] showed a maximum for Bis-GMA/UDMA/TEGDMA resin filled with 2.5 wt% Viscogel^®^ B8 (4 GPa) and Dellite 67G clay (around 3.8 GPa), andRitto et al. [46] showed a maximum for a Bis-GMA/TEGDMA matrix (8.8 GPa) enhanced with 5 wt% Cloisite 20A clay.

Mechanical strength was enhanced when dental composites were impregnated with 0.5–1 wt% clay. It is noteworthy that the maximum flexural strength was achieved for DCR1 (93.78 MPa), denoting that the utilization of just 1 wt% nanoclay efficiently supports the mechanical resistance of the dental restoratives. Under this regime, the SEM images (Figure 1) revealed adequate dispersion of nanofillers, indicating that individual clay platelets may enter the voids between the inherent inorganic particles and finally serve as supplementary filler–matrix bridges against the developed internal stresses. Most of the literature reports focus on experimental Bis-GMA/TEGDMA systems enhanced with low amounts of clay, which do not fulfill the threshold of strength (80 MPa) according to the ISO 4049 standard [54]. For instance, dental composites synthesized by Boaro et al. [34] yielded flexural strength in the range of 61.4–74.7 MPa when 2.5–10 wt% Cloisite^®^ 30B clay was added. Mahmoodian et al. [36] described nancomposite restorative resins reinforced with 1–10 wt% Cloisite^®^ 30B, which exhibited mechanical resistance between 16.6 and 47.6 MPa. Encalada-Alayola et al. [32] mentioned that the insertion of 2–10 wt% Cloisite^®^ 30B into dimethacrylate-based resins resulted in 63–78 MPa ultimate strength. It is noteworthy that the above resin formulations lack filler microparticles. Moreover, Menezes et al. [42] reported dental composites containing 2.5–10 wt% Dellite^®^ 67G or Viscogel^®^ B8 clay along with boron–alumina silicate glass microfillers, which exhibited 80–140 MPa strength. In the framework of this study, a commercially available microhybrid composite restorative, already containing silanated barium–aluminum–borosilicate glass fillers, was further reinforced with clay nanofillers, and the synthesized DCRs containing up to 3 wt% organoclay were found to meet the standard requirements (Figure 9b). Therefore, the coexistence of high-level microparticles and low-level nanoclay fillers can improve packing density and help maintain the ultimate mechanical resistance of the produced DCR. This present study also took advantage of the hydroxy groups in the quaternary ammonium modifier of clay, which are capable of hydrogen bonding with the polymer matrix to sustain the final strength. Higher nanoclay mass fractions (3–5 wt%) resulted in a gradual weakening of the composite, which was significantly greater for 5 wt% clay (*p* < 0.05). Based on the SEM observations, it can be assumed that the suggested abnormal distribution of inorganic particles within the polymer network at relatively high clay filler contents (Scheme 1) may affect the packing density of DCR and subsequently accelerate the crack propagation under the external mechanical loading.

### 3.7. Limitations of the Study

Despite the fact that most of the toxicology studies have not directly associated clay nanoparticles with toxicity or persistent inflammation, factors such as clay concentration, dispersion stability, surface charge, ion exchange capacity, particle size, and morphology [11], as well as the organomodifier type, may have relative importance to the induction of cytotoxic effects. In addition, dental restorative resins may potentially pose toxicity risks dealing with the elution of unreacted monomers and additives capable of promoting viability loss, oxidative stress, the depletion of glutathione, and the induction of apoptosis in cells, and hence, there is still a need for further investigations [77,78]. The aforementioned concerns underscore the necessity of recording the safety profile of the presented DCRs in future research studies by executing in vitro cell compatibility and cytotoxicity tests.

Herein, the specific methods used to prepare the unset dental composite pastes (e.g., hand spatulation, ambient temperature, dark conditions, and ultrasonication) constitute a combination of techniques applied either in previous research works [30,33,36,38,40,48] or in the large-scale production of dental restoratives. Nevertheless, dental dentistry utilizes robust vacuum mixers producing extremely high shear stresses that can ensure efficient homogenization of resin components in a single-step procedure. The capability of applying the above technical approach in this present study could have a higher impact on the final stability of the synthesized dental composite resins concerning their polymerization strain, solubility, and ultimate strength.

## 4. Conclusions

Dental composite resins were created by adding small amounts of clay nanoparticles to a commercially available universal microhybrid restorative material. The mechanical and physicochemical properties of the resulting composites were assessed, with particular focus on how the clay nanofillers interacted with the inherent components of the restorative matrix. XRD spectra showed predominantly exfoliated clay structures across all composites, regardless of nanoclay content. Increasing clay amounts led to lower light transmittance and greater water absorption. Despite this, the modified composites demonstrated an improved degree of conversion, while polymerization shrinkage and solubility were decreased compared to the unmodified material. The highest degree of conversion was observed in composites containing 0.5–1 wt% nanoclay, whereas 1 wt% clay resulted in the lowest polymerization shrinkage, along with the highest flexural modulus and mechanical strength. Overall, the findings suggest that existing commercial dental restorative formulations can be further refined by incorporating low levels of organically modified clay nanofillers, enhancing their mechanical and physicochemical properties. Such modifications, when incorporated into future studies on microhardness, compressive strength, in vitro cell compatibility, and cytotoxicity, could provide a more holistic approach to the development of advanced dental materials better aligned with the needs of modern clinical practice.

## Figures and Tables

**Table 1:** Characteristics of the commercially available microhybrid dental composite resin and the organomodified clay nanoparticles used in the present study.

Product/Lot	Composition	Manufacturer
Spectrum^®^ Universal Microhybrid Composite Restorative	Urethane-modified Bis-GMA resin Silanated barium–aluminum–borosilicate glass Fumed silica Camphorquinone (CQ) Ethyl-4(dimethylamino)benzoate Butylated hydroxy toluene (BHT) UV stabilizer Fluorescent agent Iron oxide pigments Titanium dioxide Shade: Opaque-A3.5	Dentsply DeTrey GmbH, Konstanz, Germany
Nanomer^®^ I.34MN	Modified clay containing 25 to 30 wt % methyl dihydroxyethyl hydrogenated tallow ammonium ion, ≤20 μm	

**Table 2:** Modified dental composite resins according to their specific composition.

Code Name	Composite
DCR0	Spectrum O-A3.5
DCR0.5	Spectrum O-A3.5 + 0.5 wt% Nanomer^®^ I.34MN
DCR1	Spectrum O-A3.5 + 1 wt% Nanomer^®^ I.34MN
DCR3	Spectrum O-A3.5 + 3 wt% Nanomer^®^ I.34MN
DCR5	Spectrum O-A3.5 + 5 wt% Nanomer^®^ I.34MN

**Table 3:** *t*-test results for equality of DC (%) and strain (%) means between different light-curing time groups at constant amount of clay nanoparticles.

Composite	DC (%)	Strain (%)		
	20 s vs. 30 s	20 s vs. 30 s	20 s vs. 5 min	30 s vs. 5 min
	*p*-Values *		*p*-Values *	
DCR0	0.819	0.037	<0.001	0.002
DCR0.5	0.412	0.008	<0.001	0.007
DCR1	0.671	0.334	0.046	0.247
DCR3	0.452	0.051	<0.001	0.033
DCR5	0.830	0.108	0.016	0.230

* The mean difference is significant at the *p* = 0.05 level.

**Table 4:** Fit parameters (*y*_0_, *a*, *b*, *c*, *d*) of the exponential sum function, coefficient of determination (*R*^2^), and theoretically calculated DC (%) values for the studied composite resins.

Composite	*y*_0_	*a*	*b*	*c*	*d*	*R* ^2^	DC (%)
DCR0	3.41 × 10^−11^	14.3308	0.1161	33.2407	101.5551	0.9974	47.57
DCR0.5	−1.56 × 10^−11^	13.6818	0.0797	37.4654	81.14512	0.9993	51.15
DCR1	1.90 × 10^−17^	17.0342	0.1217	33.1415	48.55665	0.9988	50.18
DCR3	2.94 × 10^−15^	18.5588	0.1651	30.8001	416.4168	0.9973	49.36
DCR5	1.55 × 10^−12^	11.0663	0.0722	37.5229	213.8214	0.9976	48.59

## Data Availability

The original contributions presented in this study are included in the article. Further inquiries can be directed at the corresponding author.

## Abbreviations

The following abbreviations are used in this manuscript:

ATR–FTIR	Attenuated total reflection–Fourier transform infrared
BHT	Butylated hydroxy toluene
Bis-GMA	2,2-Bis[p-(2′-hydroxy-3′-methacryloxypropoxy)phenylene]propane/Bisphenol A-glycidyl methacrylate
CHX	Chlorhexidine
CPC	Cetylpyridinium chloride
CQ	Camphorquinone
DC	Degree of monomer conversion
DCR	Dental composite resin
EDS	Energy dispersive X-ray spectroscopy
GICs	Glass ionomer cements
IQR	Interquartile range
LED	Light-emitting diode
LVDT	Linear variable displacement transducer
MMT	Montmorillonite
PMMA	Poly(methyl methacrylate)
QAIs	Quaternary ammonium ions
QPEIs	Quaternary polyethyleneimine nanoparticles
SD	Standard deviation
SEM	Scanning electron microscopy
TEGDMA	Triethylene glycol dimethacrylate
TTEGDMA	Tetraethylene glycol dimethacrylate
UDMA	Urethane dimethacrylate
XRD	X-ray diffraction spectroscopy
